# Distinct patterns of the histone marks associated with recruitment of the methionine chain-elongation pathway from leucine biosynthesis

**DOI:** 10.1093/jxb/eru440

**Published:** 2014-11-26

**Authors:** Ming Xue, Jingcheng Long, Qinlong Jiang, Minghui Wang, Sixue Chen, Qiuying Pang, Yan He

**Affiliations:** ^1^National Maize Improvement Center of China, Beijing Key Laboratory of Crop Genetic Improvement, China Agricultural University,Beijing 100193, China; ^2^Computational Biology Service Unit, Cornell University, Ithaca, NY14853, USA; ^3^Department of Biology, Genetics Institute, and Plant Molecular & Cellular Biology Program, University of Florida, Gainesville, FL 32611, USA; ^4^Alkali Soil Natural Environmental Science Center, Key Laboratory of Saline-alkali Vegetation Ecology Restoration in Oil Field, Northeast Forestry University, Harbin, Heilongjiang 14850, China

**Keywords:** *Arabidopsis*, ChIP, glucosinolate, H3K4me2, H3K4m3.

## Abstract

Aliphatic glucosinolates (GLSs) are derived from chain-elongated methionine produced by an iterative three-step process, known to be evolutionarily recruited from leucine biosynthesis. The divergence of homologous genes between two pathways is mainly linked to the alterations in biochemical features. In this study, it was discovered that a distinct pattern of histone modifications is associated with and/or contributes to the divergence of the two pathways. In general, genes involved in leucine biosynthesis were robustly associated with H3k4me2 and H3K4me3. In contrast, despite the considerable abundances of H3K4me2 observed in some of genes involved in methionine chain elongation, H3K4me3 was completely missing. This H3K4m3-depleted pattern had no effect on gene transcription, whereas it seemingly co-evolved with the entire pathway of aliphatic GLS biosynthesis. The results reveal a novel association of the epigenetic marks with plant secondary metabolism, and may help to understand the recruitment of the methionine chain-elongation pathway from leucine biosynthesis.

## Highlight

Co-ordinated with the entire pathway of aliphatic glucosinolate biosynthesis, distinct patterns of epigenetic marks are associated with the recruitment of the methionine chain-elongation pathway from leucine synthesis.

## Introduction

Glucosinolates (GLSs), once collectively referred to as mustard oil glucosides, have long been part of human life via conferring distinct flavour and aroma in *Brassica* vegetables (Kliebenstein *et al.*, 2005; Grubb and Abel, 2006). In the past decades, the importance of these sulphur-containing secondary metabolites has drawn great attention due to their roles in cancer prevention and characteristic involvement in plant defence against aphids, bacteria, and fungi (Hecht, 2000; Bednarek *et al.*, 2009; Clay *et al.*, 2009). Meanwhile, extensive studies in the model plant *Arabidopsis thaliana* have led to the nearly complete elucidation of the biosynthetic pathway, the identification of major transcriptional regulators, and the discovery of evolutionary links to other metabolic pathways (Grubb and Abel, 2006; Halkier and Gershenzon, 2006; Hirai *et al.*, 2007; Yan and Chen, 2007; He *et al.*, 2009; Sawada *et al.*, 2009; Sonderby *et al.*, 2010*a*).

According to their distinct precursor amino acids, GLSs are classified into three major types, aliphatic, indolic, and aromatic (Halkier and Gershenzon, 2006). Aliphatic GLSs are derived from methionine and represent the most abundant group in *Arabidopsis* (Grubb and Abel, 2006). The biosynthetic pathway of aliphatic GLSs proceeds in three separate phases. First, methionine undergoes a sequential addition of methylene groups to form chain-elongated derivatives (Field *et al.*, 2004; Textor *et al*., 2004 2007; Schuster *et al.*, 2006; Knill *et al.*, 2008 2009; He *et al.*, 2009; Sawada *et al.*, 2009; Redovnikovic *et al.*, 2012). Secondly, the derivatives enter the pathway to form the core skeleton of primary GLSs (Chen *et al*., 2003; Naur *et al.*, 2003; Hansen *et al.*, 2007; Geu-Flores *et al.*, 2011; Douglas Grubb *et al.*, 2014). Lastly, primary GLSs are further modified by various secondary side chain conversions (Kliebenstein *et al*., 2001 2007; Neal *et al.*, 2010; Lee *et al.*, 2012).

**Fig. 1.**
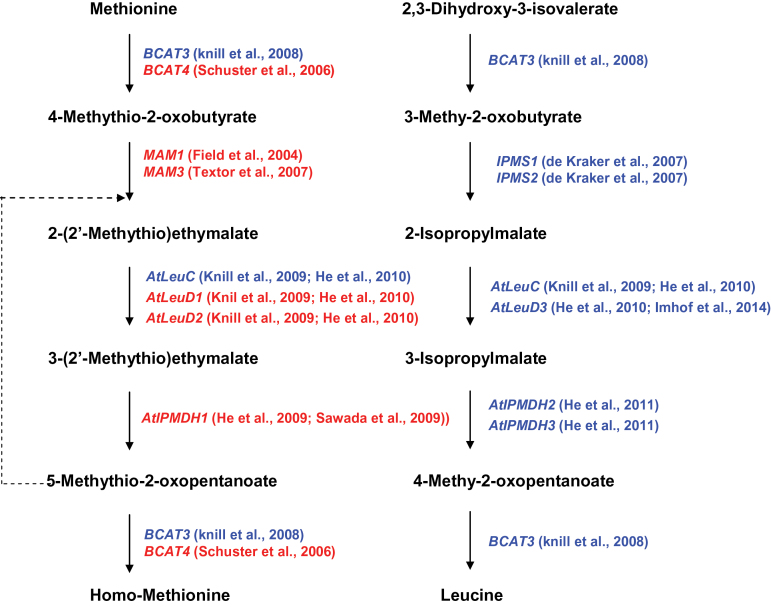
Metabolic parallels between methionine chain elongation in aliphatic GLS (left) and the late steps of leucine biosynthesis (right). The dotted line indicates the repeated cycles to generate up to six methylene resdues. The genes specified in the methionine chain-elongation pathway are highlighted in red. In comparison, the genes functional in leucine biosynthetic pathway are highlighted in blue. The representative studies referring to the characterization of those genes are shown.

The cycles of methionine chain elongation consist of four enzymatic steps, namely condensation, isomerization, oxidative decarboxylation, and deamination (Fig. 1). Interestingly, a similar cycle of 2-oxo acid-based chain-elongation reactions is also utilized in leucine biosynthesis. More importantly, genes participating in each step of the two pathways are evolutionary homologues (Fig. 1). At the condensation step, two methylthioalkylmalate synthases (MAM1 and MAM3) share ~60% amino acid identity with the two isopropylmalate synthases (IPMS1 and IPMS2) (Textor *et al*., 2004 2007; de Kraker *et al.*, 2007). The isomerization step is catalysed by isopropylmalate isomerases (IPMIs), which exist as heterodimeric enzymes and each consists of a large subunit encoded by a single gene, named *LeuC*, and a small subunit encoded by one of three genes, named *LeuD1*, *LeuD2*, and *LeuD3*. *LeuC* has dual functions in both pathways, while the functions of different *LeuD* genes are diversified, with *LeuD1* and *LeuD2* functional in methionine chain elongation, whereas *LeuD3* is functional in leucine biosynthesis (Knill *et al.*, 2009; Sawada *et al.*, 2009; He *et al.*, 2010; Imhof *et al.*, 2014). Similarly, in the oxidative decarboxylation step, isopropylmalate decarboxylase 1 (*IPMDH1*) works in methionine chain elongation, while *IPMDH2* and *IPMDH3* are involved in leucine biosynthesis (He *et al.*, 2009 2011*a*; Sawada *et al.*, 2009). Compared with the other steps, genes encoding branched-chain aminotransferases (BCATs) are relatively complicated due to the existence of six functional BCATs in the *Arabidopsis* genome. *BCAT1* and *BCAT2* are involved in the catabolism of branched-chain amino acids (Angelovici *et al.*, 2013); *BCAT3* has dual functions in both pathways (Knill *et al.*, 2008); *BCAT4* is exclusively involved in methionine chain elongation (Schuster *et al.*, 2006); while the functions of *BCAT5* and *BCAT6* are still unclear (Angelovici *et al.*, 2013). Taken together, a close relationship between the two pathways suggests that they are derived from a common ancestral pathway; or, more probably, the methionine chain-elongation pathway is evolutionarily recruited from leucine biosynthesis.

Previous studies have shown that the divergent functions of gene homologues in the two pathways could be mainly explained by differences in biochemical features (de Kraker and Gershenzon 2011; He *et al.*, 2011*b*). In this study, the patterns of distinct histone marks associated with or probably responsible for the functional diversification of gene homologues have been surveyed. It was demonstrated that the methionine chain-elongation pathway was primarily depleted of H3K4me3, a pattern in striking contrast to its robust appearance in leucine biosynthesis. The evolutionary forces driving the occurrence of such a divergence are discussed.

## Materials and methods

### Plant material and growth conditions

*Arabidopsis thaliana* (L.)Heynh., ecotype Colombia (Col-0), was used in all the experiments. Seeds were surface-sterilized and germinated on half-strength Murashige and Skoog (1/2 MS) agar medium containing 1% sucrose, and grown in a growth chamber with a photoperiod of 16h light at 22 °C and 8h dark at 20 °C for 7 d. Seedlings were transferred to soil and grown under the same conditions.

### Chromatin immunoprecipitation (ChIP) experiments

ChIP was performed as described previously, with minor modifications (He *et al.*, 2013). Briefly, ~2g of 3-week-old rosette leaves were harvested and cross-linked in 1% formaldehyde by vacuum for 10min. Then cross-linking was quenched by adding 0.125mM glucine and finally leaves were thoroughly washed three times with sterile deionized water. The materials were ground into a fine power and homogenized in buffer A [10mM TRIS-HCl pH 8.0, 0.4mM sucrose, 10mM MgCl_2_, 1mM phenylmethylsulphonyl fluoride (PMSF), 5mM β-mercaptoethanol, 1 tablet of complete Protease Inhibitor Cocktail (Roche, Penzberg, Germany)] for 20min at 4 °C with gentle shaking. The suspension was filtered into a new 50ml conical tube through two layers of Miracloth (EMD Millipore, Billerica, MA, USA), placed in a plastic funnel, and centrifuged at 4000rpm for 20min at 4 °C. The pellet was resuspended in 1ml of buffer B (10mM TRIS-HCl pH 8.0, 0.25M sucrose, 10mM MgCl_2_, 1% Triton X-100, 1mM PMSF, 5mM β-mercaptoethanol, 1 μg ml^–1^ of complete Protease Inhibitor Cocktail) and centrifuged at 14 000rpm for 10min. The pellet was resuspended in 650 μl of buffer C (10mM TRIS-HCl pH 8.0, 1.7M sucrose, 2mM MgCl_2_, 0.15% Triton X-100, 1mM PMSF, 5mM β-mercaptoethanol, 1 μg ml^–1^ complete Protease Inhibitor Cocktail), placed on top of 650 μl of a buffer C cushion, and centrifuged at 14 000rpm for 1h at 4 °C. The nuclear pellet was resuspended in 500 μl of buffer D (50mM TRIS-HCl pH 8.0, 10mM EDTA, 1% SDS, 1mM PMSF, 1 μg ml^–1^ complete Protease Inhibitor Cocktail) to release chromatin.

Chromatin was sonicated into fragments with a length of 200–300bp and centrifuged at 14 000rpm for 5min at 4 °C. The supernatant was diluted 10-fold with dilution buffer (16.7mM TRIS-HCl pH 8.0, 1.2mM EDTA, 167mM NaCl, 1.1% Triton X-100, 1mM PMSF, 1 μg ml^–1^ complete Protease Inhibitor Cocktail) and pre-cleared with Dynabeads Protein A (Invitrogen). The pre-cleared chromatin (10 μl) was saved as the chromatin input control sample. The remaining chromatin was incubated with 10 μl of antibodies (anti-H3K4me2, Millipore 07-030; anti-H3K4me3, Millipore 04-745) overnight at 4° C, and then captured by Dynabeads Protein A. After incubation, beads were washed five times in the following buffers: (i) low salt wash buffer (20mM TRIS-HCl pH 8.0, 2mM EDTA, 150mM NaCl, 0.1% SDS, 1% Triton X-100); (ii) high salt wash buffer (20mM TRIS-HCl pH 8.0, 2mM EDTA, 500mM NaCl, 0.1% SDS, 1% Triton X-100); (iii) LiCl buffer (10mM TRIS-HCl pH 8.0, 1mM EDTA, 250mM LiCl, 1% NP-40, 1% sodium deoxycholate); and (iv) TE buffer, twice (10mM TRIS-HCl pH 8.0, 1mM EDTA). Chromatin was eluted from beads with 200 μl of elution buffer (50mM TRIS-HCl pH 8.0, 10mM EDTA, 200mM NaCl, 1% SDS), treated with RNase for 2h at 3 7°C, deproteinized with proteinase K for 2h at 45 °C, and de-cross-linked at 65 °C for 8h. DNA was purified using MinElute PCR Purification Columns (Qiagen).

### Quantitative real-time PCR analysis (qPCR)

qPCR was performed using iTaq Universal SYBR Green Supermix (Bio-Rad). PCRs were performed in a 7500 Real-Time PCR machine (Applied Biosystems) according to the manufacturer’s instructions. To calculate the ChIP enrichment, input chromatin samples were defined as 100, and each ChIP sample was compared with its corresponding input. Data are presented as percentages of input DNA. All data were averages of three independent experiments. Primer sequences used for qPCR are listed in Supplementary Table S2 available at *JXB* online.

### Total RNA extraction and reverse transcription–qPCR

Total RNA was extracted with TRIzol (Invitrogen Inc.) from the same 3-week-old rosette leaf samples that were used for the ChIP experiments. RNA was reverse transcribed with SuperScript III reverse transcriptase (Invitrogen). cDNA was analysed by qPCR. *ACTIN7* (AT5G09810) was used as an internal standard to normalize the relative expression levels. Gene-specific primers were designed and are listed in Supplementary Table S2 at *JXB* online. All data are shown as the average of three independent experiments.

### Computational analysis

The ChIP-Seq data sets for various histone modifications were obtained from the NCBI Short Read Archive (SRA) and aligned to the *Arabidopsis* genome (TAIR10) as previously described (Ha *et al.*, 2011). The position of the transcription start site (TSS) was defined as the first base of the transcript. Composite plots were generated by averaging values in each of 50bp sliding windows.

## Results

### Comprehensive *in silico* analysis revealed a distinct pattern of histone modifications

**Fig. 2.**
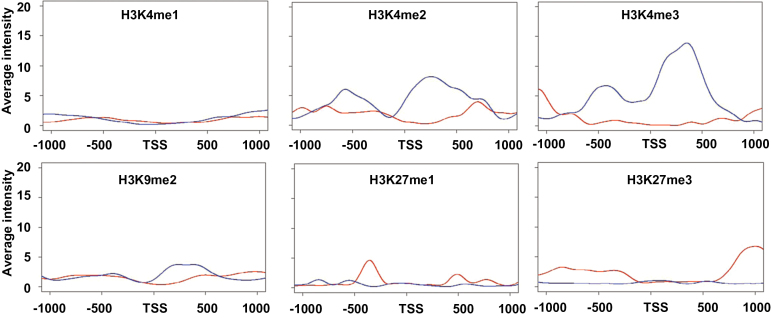
The composite plot shows the distribution of histone marks around the transcription start site (TSS; ±1kb). The red line depicts genes specifically involved in the methionine chain-elongation pathway, including *MAM1*, *MAM3*, *LeuD1*, *LeuD2*, *IPMDH1*, and *BCAT3*. The blue line depicts genes functional in leucine biosynthesis, including *IPMS1*, *IPMS2*, *LeuC*, *LeuD3*, *IPMDH2*, *IPMDH3*, and *BCAT3*.

Post-translational modifications of histone play crucial roles in maintaining normal transcription levels and patterns by directly or indirectly shaping the structural properties of the chromatin. To explore the distinct association with certain types of histone modification at the pathway level, occurrences of six chromatin marks representing the active chromatin state (H3K4me, H3K4me2, and H3K4me3) and the repressed chromatin state (H3K9me2, H3K27me1, and H3K27me3) were examined and the results were compiled into two sets, with the first focusing on methionine chain elongation and the second on leucine biosynthesis. Data on histone modifications were obtained from publically available *Arabidopsis* data sets (Supplementary Table S1 a*t JXB* online). The intensity of histone marks was counted using a 50bp sliding window around the TSS (± 1kb). As shown in Fig. 2, of the investigated marks, the patterns of two active marks, H3K4me2 and H3K4me3, exhibited striking differences, with a substantial presence in leucine biosynthesis and a low occurrence in the methionine chain-elongation pathway. In contrast, none of the repressed marks showed significant differences between the two pathways.

**Fig. 3.**
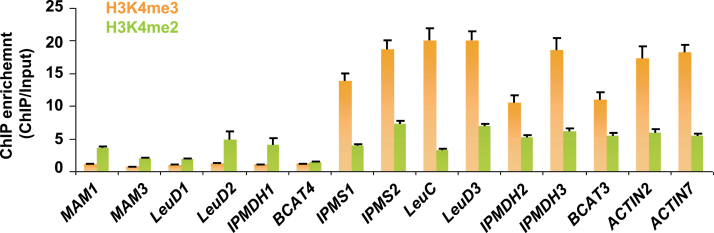
Levels of H3K4m2 and H3K4me3 for genes in the leucine and methionine chain-elongation pathways determined by ChIP-qPCR analysis. The *y*-axis shows the fold ChIP enrichment over input. Two housekeeping genes under the regulation of H3K4me3 as reported previously were used as positive controls (Saleh *et al.*, 2008; Jang *et al.*, 2011). The data were from three biological replicates and are shown as mean values ±SD. (This figure is available in colour at *JXB* online.)

To validate the patterns of the H3K4me2 and H3K4me3 marks revealed by *in silico* analysis, the occurrences of two marks at each individual gene locus were experimentally checked by performing ChIP coupled with qPCR (ChIP–qPCR). As shown in Fig. 3, the H3K4me3 mark was hardly detected for all the genes in the methionine chain-elongation pathway, while, in comparison, it was widely observed in the genes functional in leucine biosynthesis. In contrast to the complete absence of H3K4me3, the H3K4me2 mark was detectable in some of the genes in the methionine chain-elongation pathway but to a lesser extent than in leucine biosynthesis (Fig. 3). Therefore, it is concluded that the recruitment of the methionine chain-elongation pathway from leucine biosynthesis was accompanied by distinct patterns of H3K4me2 and H3K4me3 marks.

**Fig. 4.**
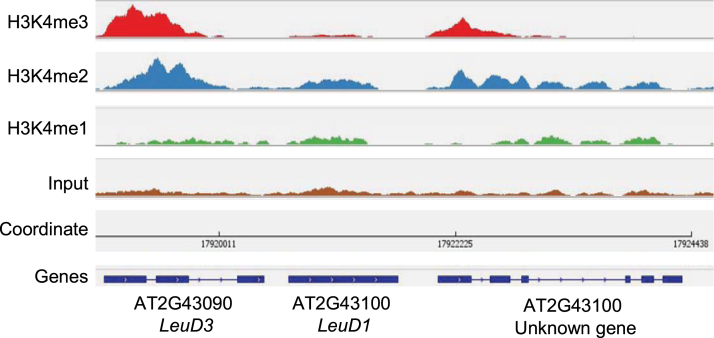
H3K4me1, H3K4me2, and H3K4me3 signal tracks in the *LeuD1* and *LeuD3* locus. The *y*-axis of each profile represents the number of reads at each position normalized by the total number of reads in a given data set. Levels of H3K4m2 and H3K4me3 were determined by ChIP-qPCR analysis. (This figure is available in colour at *JXB* online.)

The landscape of H3K4me2 and H3K4me3 in *Arabidopsis* has shown that both marks are predominantly absent from pericentromeric heterochromatin regions (Zhang *et al.*, 2009). However, the random distributions of genes in both pathways on the genome rule out the likelihood that the absence of H3K4me3 marks in the methionine chain-elongation pathway was related to chromosomal localization (data not shown). In particular, an extreme case is shown in Fig. 4, where *LeuD1* and *LeuD3* are tandemly arranged on chromosome 2 in a head-to-tail manner while the levels of H3K4me2 and H3K4me3 behaved profoundly differently in the two genes.

### A lack of H3K4me3 was not accompanied by decreased gene expression

**Fig. 5.**
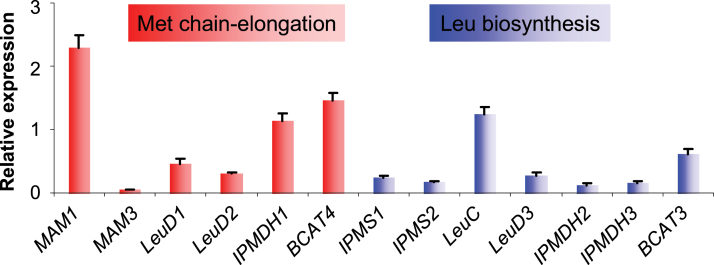
Relative gene expression levels in the same samples used in ChIP analysis. The values were normalized using *ACTIN7* as an internal reference. The data were from three biological replicates and are shown as mean values ±SD. Note that the method of relative expression, not absolute quantification, was used in the study as the amplification efficiencies of primer pairs are comparable with each other (Supplementary Table S1). (This figure is available in colour at *JXB* online.)

It has been well established that association with H3K4me2 and H3K4me3 marks reflects the active state of gene transcription (Zhang *et al.*, 2009). To elucidate the scenario whereby the low levels of H3K4me2 and H3K4me3 may lead to a reduction in gene transcription, the expression of each gene in the two pathways was examined using RT–qPCR analysis. As shown in Fig. 5, in general, the levels of gene expression in the methionine chain-elongation pathway did not deviate from those in leucine biosynthesis. More importantly, for each individual gene, the correlation of H3K4me3 abundance and gene expression was not observed (Fig. 5). For instance, the expression levels of *MAM1* and *IPMDH1* were significantly higher than those of *IPMS1/IPMS2* and *IPMDH2/IPMDH3*, respectively. These results indicate that the low level of H3K4me2 and the complete lack of H3K4me3 have no effect on gene expression. Also, the state of gene transcription could not be attributed to the low occurrence of H3K4me2 and H3K4me3.

### The H3K4me3-depleted pattern persistently associated with aliphatic GLS biosynthesis and transcriptional regulation

**Fig. 6.**
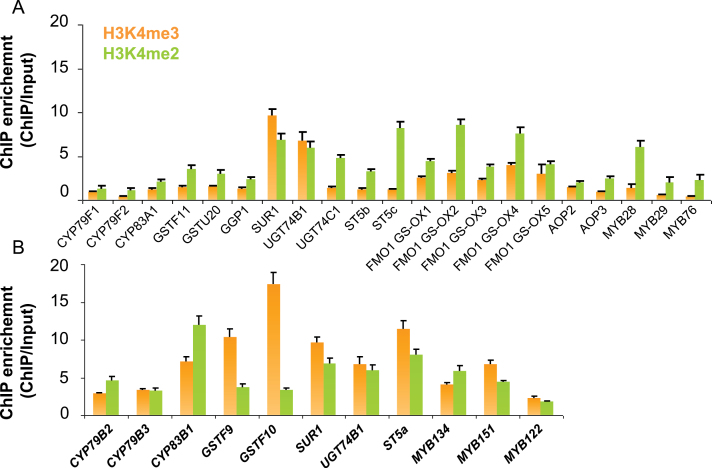
Levels of H3K4m2 and H3K4me3 for genes in the aliphatic (A) and indolic (B) GLS pathway were determined by ChIP-qPCR analysis. The *y*-axis shows the fold ChIP enrichment over input. The data were from three biological replicates and are shown as mean values ±SD. (This figure is available in colour at *JXB* online.)

The end-products of the methionine chain-elongation pathway serve as the precursors entering aliphatic GLS biosynthesis. To test the likelihood that the absence of H3K4me3 represents a common character co-ordinated in the aliphatic GLS biosynthetic pathway, the levels of H3K4me2 and H3K4me3 were assayed for all the genes involved in the formation of the aliphatic GLS core structure. As shown in Fig. 6A, no detectable H3K4me3 could be identified in most of these genes except for *SUR1* and *UGT74B1*, two genes playing roles in both aliphatic and indolic GLS pathways. In contrast, a moderate level of occurrence of H3K4me2 was found in most of the genes (Fig. 6A). Three transcription factors (TFs), MYB28, MYB29, and MYB76, have been reported as master regulators modulating aliphatic GLS biosynthesis (Gigolashvili *et al*., 2007 2008; Hirai *et al.*, 2007; Sonderby *et al.*, 2007 2010*b*; Li *et al.*, 2013). Previous studies have shown that these TFs were synergistically co-regulated with other biosynthetic genes at the transcriptional level (Hirai *et al.*, 2007). Here it was found that the H3K4me3-depleted pattern was also conserved in these TFs (Fig. 6A), indicating that the genes involved in aliphatic GLS biosynthesis and the corresponding transcriptional regulation were co-ordinated at both the genomic and transcriptional level.

To examine whether the absence of H3K4me3 marks occurs specifically in the genes for the aliphatic GLS pathway, the levels of H3K4me2 and H3K4me3 in the genes for the indolic GLS pathway were assessed. As shown in Fig. 6B, substantial amounts of H3K4me3 were detected in most of genes involved in the indolic GLS pathway, suggesting that the absence of the H3K4me3 epigenetic mark was evolutionarily relevant to the divergence of aliphatic and indolic GLSs.

To test the possibility that the disappearance of the H3K4me3 mark may symbolize a universal event during the evolution of a secondary metabolic pathway from primary metabolism, the occurrence of the H3K4me3 mark was examined by *in silico* analysis in genes involved in three other groups of plant secondary metabolism, namely sterol, lignin, and terpene. As shown in Supplementary Fig. S1A at *JXB* online, substantial amounts of H3K4me3 were observed in all the pathways investigated, implying that the diversification of genes into secondary metabolism would not account for the depleted pattern of the H3K4me3 mark in GLS synthesis.

It has been reported that in contrast to nitrogen metabolism, the nearly complete sulphur assimilation and GLS pathways are preferentially abundant in the bundle sheath (BS) cells of C_3_ plants (Aubry *et al.*, 2014). To investigate the possible relevance of BS localization to the specific H3K4me3-depleted pattern in GLS biosynthesis, the levels of H3K4me3 in the genes involved in the sulphur and nitrogen assimilation pathways were checked. As shown in Supplementary Fig. S1B at *JXB* online, similar to nitrogen metabolism, genes involved in the sulphur assimilation pathway were highly linked with H3K4me3, suggesting that the depleted pattern of the H3K4me3 mark could not be attributed to the BS localization of GLS synthesis.

## Discussion

To better elucidate the origin of such diversity, it is intriguing to understand the evolution of secondary metabolite pathways (de Kraker and Gershenzon, 2011). Primarily based on phylogenetic analyses and analogous biochemical characterization, the methionine chain-elongation pathway is believed to have evolved from leucine biosynthesis (He *et al.*, 2009; de Kraker and Gershenzon, 2011). This recruitment is in part fulfilled by the adapted enzyme catalytic ability. deKraker and Gershenzon (2011) demonstrated that a combination of the loss of the regulatory domain and a few additional amino acid exchanges can convert IPMS to MAM. In addition, the small subunit of the heteromeric IPMI was specialized into each pathway (He *et al.*, 2009; Knill *et al.*, 2009; Imhof *et al.*, 2014). Furthermore, it is demonstrated that a single amino acid substitution has the ability to interchange the substrate specificity of three IPMDHs (He *et al.*, 2011*a*). Moreover, *BCAT3* is functional in both pathways while *BCAT4* is specialized in the methionine-chain elongation pathway (Schuster *et al.*, 2006; Knill *et al.*, 2008). Taken together, all these studies indicate that the evolutionarily driven alterations in amino acid sequences play critical roles in shaping the functional divergences among gene homologues between the two pathways. However, whether epigenetic variation plays a role or, alternatively, co-evolvution of such an adaption, has never been explored previously.

Genome-wide patterning of H3K4me, H3K4me2, and H3K4me3 has identified that approximately two-thirds of genes in *Arabidopsis* contain at least one type of H3K4 (Zhang *et al.*, 2009; van Dijk *et al*., 2010). In this study, it was found that genes involving the methionine chain-elongation pathway show a common H3K4me3-depleted pattern, raising three intriguing evolutionary questions. (i) How was this pattern formed. (ii) Is this pattern beneficial for gene regulation. (iii) What could be the role of different patterns, if any? Previous studies have demonstrated that H3K4me3 plays important roles in regulating gene transcription, which is reflected by an intimate association of H3K4me3 with the active state of gene transcription (Zhang *et al.*, 2009; van Dijk *et al*., 2010). However, surprisingly, the qPCR analysis conducted here has confirmed that the levels of gene expression in the methionine chain-elongation pathway are not different from those in leucine biosynthesis, indicating that the conditions of gene transcription have nothing to do with the appearance of the depletion of H3K4me3. Therefore, it still remains elusive as to how this pattern was formed. Interestingly, two recent studies have demonstrated that H3K4me3 contributes to the transcriptional memory of stress-responsive genes in *Arabidopsi*s (Ding *et al.*, 2012; Kim *et al.*, 2012). However, it is unknown whether or not a similar phenomenon could happen in GLS biosynthetic genes. Previous studies have shown that expression of GLS biosynthetic genes was induced by pathogen infection and methyl jasmonate (Gigolashvili *et al.*, 2007; Hirai *et al.*, 2007). Since all the experiments in this study were conducted under ambient conditions, the likelihood that H3K4me3 plays roles in the induction of GLS biosynthesis and the accompanying transcriptional memory after challenge by stress deserve further extensive investigation. Meanwhile, network analysis has demonstrated that along with GLS biosynthesis, genes in the methionine chain-elongation pathway show strong co-regulation at the transcriptional level. It is proposed that such a scheme for H3K4me3 depletion may be directly or indirectly responsible for the gene co-expression network through the creation of a flexible histone status.

In summary, it is reported that other than the changes in gene sequences, the ‘epigenetic code’ was utilized in the recruitment of the methionine chain-elongation pathway from leucine biosynthesis. In addition, this involvement appears to be independent from gene transcription, while it co-evolved within the entire GLS biosynthesis. Since this is the first study on the direct interplay between histone modification and secondary metabolism, the findings have broad implications for how secondary metabolism was recruited from primary metabolism and add a new epigenetic dimension to the regulation of GLS biosynthesis.

## Supplementary data

Supplementary data are available at *JXB* online.

Figure S1. Composite plot showing the distribution of the H3K4me3 mark around the transcription start site (±1kb) in representative secondary and primary metabolism pathways.

Table S1. List of genome-wide histone modification data sets used in the study.

Table S2. Primer sequences used in the study.

Table S3. List of genes used in the analysis of Fig. S1.

Supplementary Data
