# Supplementary material for: Distinct patterns of the histone marks associated with recruitment of the methionine chain-elongation pathway from leucine biosynthesis
Source: J Exp Bot. 2014 Nov 26;66(3):805–12. doi: 10.1093/jxb/eru440 (PMC4321544; doi:10.1093/jxb/eru440)
Supplement: Supplementary Data [file supp_66_3_805__index.html]

Distinct patterns of the histone marks associated with recruitment of the methionine chain-elongation pathway from leucine biosynthesis — Distinct patterns of the histone marks associated with recruitment of the methionine chain-elongation pathway from leucine biosynthesis — Supplementary Data 

# Distinct patterns of the histone marks associated with recruitment of the methionine chain-elongation pathway from leucine biosynthesis

## Supplementary Data

Data files

**Files in this Data Supplement:**

- Supplementary Data - Supplementary Data
