# Supplementary material for: Distinct patterns of the histone marks associated with recruitment of the methionine chain-elongation pathway from leucine biosynthesis
Source: J Exp Bot. 2014 Nov 26;66(3):805–12. doi: 10.1093/jxb/eru440 (PMC4321544; doi:10.1093/jxb/eru440)
Supplement: Supplementary Data [file supp_eru440_jexbot134254_file001.pdf]

Distinct patterns of histone mark associated with recruitment of methionine chain-elongation pathway from leucine biosynthesis

Ming Xue, Jincheng Long, Qinlong Jiang, Minghui Wang, Sixue Chen, Qiuying Pang, and Yan He

SUPPLEMENTARY DATA

Supplementary Figure S1. Composite plot shows distribution of H3K4me3 mark around TSS (±1kb) in representative secondary (A) and primary (B) metabolism pathways. Genes used in the analysis are listed in Table S3. TSS: transcription start sites.

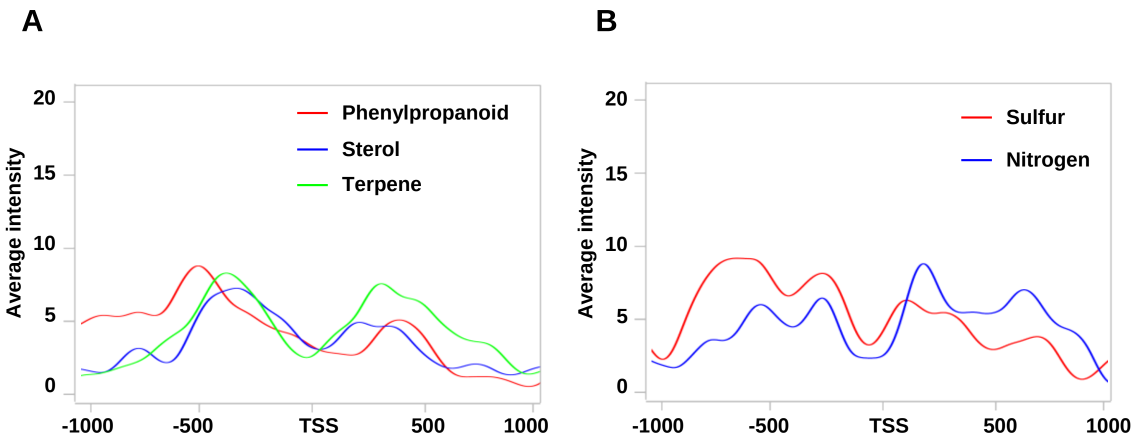

Supplementary Table S1. List of genome-wide histone modification datasets used in the study

Accession number	Citation
GSE28398	Luo et al., 2012
GSE22276	Ha et al., 2011
GSM624616	Lu et al., 2011
GSM925660	Moissiard et al., 2012
GSE11657	van Dijk et al., 2010

SupplementaryTable S2. Primer sequences used in the study

Primer	Sequence (5'-3')	Experiment [a,b]	Amplification efficiency [c, d]
MAM1-F	GCATTGTTCATCTCATGTTG	a, b	92%
MAM1-R	GCGGAGTGAGGGATCCACCA	a, b	
MAM3-F	CTCTCTTCGCCTGACCCGTCCA	a, b	96%
MAM3-R	ACACGCACATAGTTCTTGTGG	a, b	
LeuD1-F	CTAACGAAGCCCTAGCCAACA	a, b	96%
LeuD1-R	TCCTGGCTCAATGAACCGTG	a, b	
LeuD2-F	ACGATCATCACACGCGTCGCT	a, b	91%
LeuD2-R	GGTAAGCCGTTAAGCGCGAA	a, b	
IPMDH1-F	CGAACATCAGTCTGAATGCGA	a, b	101%
IPMDH1-R	CGATACCGTCTCCGGGAAGC	a, b	
BCAT4-F	GCGAATGTCAAGTGGGAAGAG	a, b	96%
BCAT4-R	TGGCCATACTGAAGAACAGCAG	a, b	
IPMS1-F	CCAACCATCTCACCACCGTTC	a, b	94%
IPMS1-R	CGAAGACGCGGACGTAGTTTGG	a, b	
IPMS2-F	CTTCACCATCGTTCGGTGTACCT	a, b	92%
IPMS2-R	GCAGCGGGAAATCCAGCTTCG	a, b	
LeuC-F1	GCAGAGTGATTTGGGGATTTC	a	n.s
LeuC-R1	TACCGATCCTGTAGTTCCAGGA	a	
LeuC-F2	CCTGGAACTACAGGATCGGTGAA	b	97%
LeuC-R2	CTCCGGATCCCAAACCTTGG	b	
LeuD3-F	CGACAACATCGACACTGACCAA	a, b	98%
LeuD3-R	CCAGTAGCAACAGAGTTCCTG	a, b	
IPMDH2-F	ATCCGGACGGTCAAGGTTCC	a, b	94%
IPMDH2-R	TTGGCAATGGAGACAACCTC	a, b	
IPMDH3-F	CATCCGTCTGGAGATCATACC	a, b	98%
IPMDH3-R	GAGAAATCCAGCTTTCTGAAGCA	a, b	
BCAT3-F	GCCTCCGTCTCCCTTATCAAAG	a, b	96%
BCAT3-R	TGATGGAGGCAAATGACG	a, b	
ACTIN2-F	CCAAGGTAATAGGAACTTTCTG	a	n.s
ACTIN2-R	GAACATGGATCTCTCCATCAAGG	a	
ACTIN7-F1	GACTTCGTGTATGCTCGATTGATC	a	n.s
ACTIN7-R1	CTGAATATCCTCACCATCGGCCA	a	
ACTIN7-F2	CCGGTATTGTGCTCGATTCTG	b	99%
ACTIN7-R2	TTCCCGTTCTGCGGTAGTGG	b	
CYP79F1-F	TGCCGGCATCCGTGCCATCAC	a	n.s
CYP79F1-R	CGCATGGTCACTGCGTAACC	a	
CYP79F2-F	GGAACCTCATCGTACGGTGAAC	a	n.s
CYP79F2-R	CCTAGTCTCCCATCATCCGAA	a	
CYP83A1-F	ACTCCGACGAGATCGCTAGAG	a	n.s
CYP83A1-R	CGTAACATGTCTCCTTCCAAAC	a	
GSTF11-F	CGCGAGCCATAGCGAGGTAC	a	n.s
GSTF11-R	CATCCAGGACCTTGTCGAAC	a	

GSTU20-F	CAGCCCAGAATCGAGCCTGA	a	n.s
GSTU20-R	ACTCCTCCAGAGTAATCCCA	a	
SUR1-F	TGAGCGAAGAACAACCACACGC	a	n.s
SUR1-R	CGACGGCGTCTTCAGCTTCG	a	
GGP1-F	GAGATACGCTCTGTTTCTAGC	a	n.s
GGP1-R	CTGATGACCAAAGCAGATGC	a	
UGT74B1-F	CTGAGTAAGTATCGACGCTG	a	n.s
UGT74B1-R	CGGAAACAACTCCCAAAGTG	a	
UGT74C1-F	GAAGCAAAGAAGGGTCACGTACTG	a	n.s
UGT74C1-R	CGGTCAGGCTTCGAGAAGTAGA	a	
ST5b-F	CCAAGGCTACGATCACGACC	a	n.s
ST5b-R	GCGATGGCGAAAGTCAGGGCT	a	
ST5c-F	CGACGTCGTCGTATCAGAGTC	a	n.s
ST5c-R	CTTTGAGCCAAGTCGTGCCGGT	a	
MYB28-F	GCCATGTTGCGTCGGAGAAGG	a	n.s
MYB28-R	GTTGCCACGAGAAGCATGAAGC	a	
MYB29-F	CAAGATGTCAAGAAAGCCATGTTG	a	n.s
MYB29-R	CTCTCCTCTCTTGATGTCAGG	a	
MYB76-F	GGAGCATGGACTACAGAAGAGG	a	n.s
MYB76-R	CACGAGATGCATGAAGCATGATG	a	
FMO GS-OX1-F	GATGAGTAGTTCCAGAGACC	a	n.s
FMO GS-OX1-R	CACAATCTGTTCGAAACACG	a	
FMO GS-OX2-F	CACAGAGATCATACGACAGTC	a	n.s
FMO GS-OX2-R	GTCGGATTCGGCTTTGGGTG	a	
FMO GS-OX3-F	GGCCTGAAGCTGATGCAAC	a	n.s
FMO GS-OX3-R	GGCTAAGTGGATCGGAATCG	a	
FMO GS-OX4-F	TGGCACCAGCTCCTAGTCCA	a	n.s
FMO GS-OX4-R	GTCGGGTCAAGACCAAGCTC	a	
FMO GS-OX5-F	GATGGCACCAGCACGAACC	a	n.s
FMO GS-OX5-R	CTTGAATGGACGATGGTTCG	a	
AOP2-F	GCACACGTTTCGTTGTTTGG	a	n.s
AOP2-R	GGGTTCATGCAGTCTTCAGC	a	
AOP3-F	GATCAAGTGTCAATGGAGCT	a	n.s
AOP3-R	TACCTGATACTCTTGTTACC	a	
CYP79B2-F	GCTAGGAACGGCGTTGACCA	a	n.s
CYP79B2-R	CTTCTGGTAACCGGAATTGACC	a	
CYP79B3-F	GGCATCTCAATGAGTGTTCG	a	n.s
CYP79B3-R	CTTCACCATTGGGTAAAGGTC	a	
CYP83B1-F	GAGTAGCTCCTTGGCTAGCT	a	n.s
CYP83B1-R	TAGTAGCGGCTGCAGCCTTC	a	
GSTF9-F	CACGAGTCGCGATTCTCTCT	a	n.s
GSTF9-R	CTGTAGAGCGAGATAAGCAG	a	
GSTF10-F	CCTACTACCGCAAACGCAGCT	a	n.s
GSTF10-R	GATACTCAGGCTGTCTCTGTTC	a	
ST5a-F	CGTCGTAACGAGAACGATTG	a	n.s

ST5a-R	CAATGGAATCAAAGACAACC	a	
MYB34-F	GACTTACTGACCGGGATCATC	a	n.s
MYB34-R	CTACATTGACCTCCGGCGAA	a	
MYB51-F	GGTCTGCTATAGCTCGTGGAC	a	n.s
MYB51-R	CTCAGACAGAACACTCTGATTG	a	
MYB122-F	GTTGTCAGGATCATCATCAGC	a	n.s
MYB122-R	GAACGTGGATGAGGATAGAG	a	

[a] used for ChIP-qPCR

[b] used for RT-qPCR

[c] The amplification efficiency (E) of the primer set used in RT-qPCR was calculated from the slope of the standard curve.

[d] n.s: not specified.

SupplementaryTable S3. List of genes used in the analysis of Figure S1.

Phenylpropanoid metabolism		Sterol metabolism		Sterol metabolism	
**Gene ID**	**Name**	**Gene ID**	**Name**	**Gene ID**	**Name**
At2g37040	*PAL1*	AT1G58440	*SQE1*	AT5G47720	*ACT1*
At3g53260	*PAL2*	AT2G22830	*SQE2*	AT5G48230	*ACT2*
At2g30490	*REF3*	AT4G37760	*SQE3*	AT4G11820	*HMGS*
AT1G51680	*At4CL1*	AT2G07050	*CAS1*	AT1G76490	*HMG1*
AT3G21240	*At4CL2*	AT5G13710	*SMT1*	AT2G17370	*HMG2*
AT5G48930	*HCT*	AT1G20330	*SMT2-1*	AT5G27450	*MK*
AT2G40890	*REF8*	AT1G76090	*SMT2-2*	AT1G31910	*PMK*
AT4G34050	*CCoAOMT1*	AT5G50375	*CPI1*	AT2G38700	*MVD1*
AT1G15950	*CCR1*	AT1G11680	*CYP51A2*	AT3G54250	*MVD2*
AT4G36220	*FAH1*	AT3G52940	*FK*	AT4G15560	*DXS1*
AT5G54160	*AtOMT1*	AT1G20050	*HYD1*	AT5G62790	*DXR*
AT3G19450	*CAD-C*			AT2G02500	*MCT*
AT4G34230	*CAD-D*			AT2G26930	*CMK*
				AT1G63970	*MDS*
				AT5G60600	*HDS*
				AT4G34350	*HDR*

Sulfur assimilation		Nitrogen assimilation	
**Gene ID**	**Name**	**Gene ID**	**Name**
AT3G22890	*APS1*	AT1G77760	*NIA1*
AT1G19920	*APS2*	AT1G37130	*NIA2*
AT4G14680	*APS3*	AT2G15620	*NIR*
AT5G43780	*APS4*	AT5G37600	*GLN1;1*
AT4G04610	*APR1*	AT1G66200	*GLN1;2*
AT1G62180	*ARP2*	AT3G17820	*GLN1;3*
AT4G21990	*ARP3*		
AT5G04590	*SIR*		
AT2G14750	*APK1*		
AT4G39940	*APK2*		
